# Transient elastography measurements of the liver and transplanted kidney in patients with AA amyloidosis: a cross-sectional comparative study

**DOI:** 10.1007/s00296-025-05906-3

**Published:** 2025-07-01

**Authors:** Murat Bektaş, Bilger Çavuş, Besim Fazıl Ağargün, İbrahim Volkan Şenkal, Nevzat Koca, Burak İnce, Selma Sarı, Ahmet Burak Dirim, Metban Güzel Mastanzade, Gizem Dağcı, Pelin Karaca Özer, Mehmet Aydoğan, Melek Büyük, Yasemin Yalçınkaya, Bahar Artım-Esen, Murat İnanç, Mine Güllüoğlu, Halil Yazıcı, Sevgi Kalayoğlu Beşışık, Selman Fatih Beşışık, Ahmet Gül

**Affiliations:** 1 Division of Rheumatology, Department of Internal Medicine, Istanbul Faculty of Medicine, Istanbul University, Turgut Özal Millet Cd, 34093 Istanbul, Fatih Turkey; 2 Division of Gastroenterology, Department of Internal Medicine, Istanbul Faculty of Medicine, Istanbul University, Istanbul, Turkey; 3 Department of Internal Medicine, Istanbul Faculty of Medicine, Istanbul University, Istanbul, Turkey; 4 Division of Nephrology, Department of Internal Medicine, Istanbul Faculty of Medicine, Istanbul University, Istanbul, Turkey; 5 Division of Hematology, Department of Internal Medicine, Istanbul Faculty of Medicine, Istanbul University, Istanbul, Turkey; 6 Department of Cardiology, Istanbul Faculty of Medicine, Istanbul University, Istanbul, Turkey; 7 Department of Pathology, Istanbul Faculty of Medicine, Istanbul University, Istanbul, Turkey; 8 Division of Rheumatology, Department of Internal Medicine, Istanbul Aydin University, Istanbul, Turkey

**Keywords:** AA amyloidosis, Elasticity imaging techniques, Familial Mediterranean Fever, Immunoglobulin light-chain amyloidosis, Kidney transplantation, Liver

## Abstract

To evaluate the utility of elasticity imaging techniques, such as transient elastography (FibroScan), in assessing the liver and transplanted kidney in patients with AA amyloidosis (AA-A). This study was conducted in patients with AA-A, while patients with immunoglobulin light chain amyloidosis (AL-A), Familial Mediterranean Fever (FMF) without amyloidosis, and healthy controls (HC) were included as comparison groups for liver stiffness (LS) measurements. Additionally, kidney stiffness (KS) was measured in renal transplant recipients (RTRs) with transplants due to AA-A or other causes of chronic renal failure. LS evaluations were performed in 65 patients with AA-A, 14 with AL-A, 20 with FMF, and 27 with HC. LS (kPa) was significantly higher [median (IQR)] in patients with AA-A [6.4 (5.4)] and AL-A [9.8 (11)] compared to HC [4.7 (1.7)] (p < 0.001). However, the difference between AL-A and AA-A was not statistically significant. LS values were comparable in FMF-AA patients with [6.8 (6.6)] and without [5.7 (3.6)] liver involvement, and FMF patients without amyloidosis [7.15 (4.6)]. The median KS values were comparable in 19 AA-A and 16 disease controls. However, KS values were significantly higher in patients with recurrent amyloidosis in the transplanted kidney [29.3 (18.9)] compared to those without recurrence [10.9 (7.7)] (p = 0.003). In our study, increased liver stiffness measurements were observed in patients with AA-A, AL-A, and FMF compared to HC. Transient elastography using FibroScan appears to be a promising non-invasive tool for assessing liver involvement in AA-A and may aid in detecting recurrence of amyloidosis in transplanted kidneys. Further studies are needed to validate the utility of FibroScan in evaluating the involvement of the liver and kidneys in patients with AA-A.

**Supplementary Information:**

The online version contains supplementary material available at 10.1007/s00296-025-05906-3.

## Introduction

Amyloidosis is a protein-folding disorder characterized by the extracellular deposition of certain misfolded precursor proteins that aggregate to form insoluble fibrils [1]. In AA amyloidosis (AA-A), serum amyloid A protein serves as the fibril precursor [2], whereas AL amyloidosis (AL-A) originates from monoclonal immunoglobulin light chains [3]. AA-A is commonly associated with uncontrolled chronic inflammatory diseases such as rheumatoid arthritis, ankylosing spondylitis, and autoinflammatory diseases such as Familial Mediterranean Fever (FMF). FMF is prevalent in Eastern Mediterranean countries and has long been recognized as the leading cause of AA-A [4].

Amyloid fibrils can be deposited in virtually all tissues and organs, and pathological investigations are the gold standard for their confirmation. However, the use of non-invasive methods to detect amyloid deposition is particularly valuable for organs that carry a higher risk of complications during biopsy, such as the liver, kidney, and heart [5, 6]. There have been efforts to develop new methods to document the extent of amyloid deposition, but none have become widely accessible in routine clinical practice. One such technique is whole-body serum amyloid P (SAP) component scintigraphy, which has proven valuable in assessing and quantifying amyloid burden in patients with both AA and AL amyloidosis [7]. In *Lachmann *et al*.*’s study, the higher amyloid burden detected by SAP scintigraphy was associated with worse outcomes, such as a higher prevalence of end-stage renal disease and increased mortality [8]. This study also showed lower mortality rates in patients with regression of amyloid deposits on SAP scintigraphy. Despite its clinical utility, SAP scintigraphy remains largely unavailable and is rarely used outside of specialized centers.

Several studies have explored alternative diagnostic techniques such as magnetic resonance elastography (MRE) [9], shear-wave elastography (SWE) [10], and transient elastography (TE) using the FibroScan system. FibroScan is an ultrasound-based, safe, fast, and easily performed method that has been used in the follow-up of several chronic liver diseases (CLD) by measuring liver stiffness (LS), which correlates with liver fibrosis across different conditions [11–13]. Although FibroScan is a useful tool for LS measurement (LSM), an increase in LS is not specific to liver fibrosis. LS can be elevated in several conditions, including heart failure [14], cholestasis [15], and accumulation of amyloid fibrils [16]. While few studies have investigated the use of FibroScan for detecting the liver involvement in AL-A, most of which consisted of case reports and small uncontrolled studies [16, 17]. Furthermore, there has been no data on the utility of FibroScan regarding the liver and transplanted kidney involvement in patients with AA-A.

In this study, we aimed to evaluate the potential utility of the FibroScan method for assessing liver and transplanted kidney involvements in patients with AA-A, with comparisons based on the biochemically defined organ involvements according to the criteria set by *Gertz *et al. [18].

## Materials and methods

### Patients and data

This cross-sectional study was conducted at a single referral rheumatology center at the Istanbul Faculty of Medicine in Türkiye, which takes care of a large cohort of patients with amyloidosis. The study population consisted of patients with biopsy-proven AA-A, and a group of patients with AL-A, FMF patients without amyloidosis, and healthy controls (HC) were included for the comparative investigation of LSM. Patients’ data were collected using a standard form based on their medical charts. AA-A patients were further divided into two groups: those with FMF (FMF-AA) and those without FMF (non-FMF-AA).

All study participants were over 18 years of age. All FMF patients exhibited typical clinical findings that met the conservative *Livneh* criteria set for diagnosis (1 major or 2 minor criteria, or 1 minor plus 5 supportive criteria) [19]. Parameters with a potential effect on LSM, including age, sex, history of diabetes mellitus (DM), and body mass index (BMI) were collected from all groups [20]. We used the following criteria to include and exclude the volunteers in the study: Inclusion criteria, a. the patients with a diagnosis of AA-A, AL-A, FMF, b. Healthy controls with no history and findings of liver disease for liver stiffness evaluation, c. Renal transplant recipients due to AA-A and other causes of kidney failure for kidney stiffness evaluation, d. Participants who are over 18 years of age and consent for stiffness evaluation; Exclusion criteria: a. Volunteers who are under 18 years, b. AL-A and AA-A patients without biopsy confirmation and those with missing data for the documentation of the type of amyloidosis, c. The participants who did not give written consent for liver and kidney stiffness evaluation, d. The patients and healthy controls who had known chronic liver disease, including chronic hepatitis, steatohepatitis, liver cirrhosis, and/or post-infection positive hepatitis B and/or C serology.

Transient elastography is not typically used to assess the kidneys; however, the relatively superficial location of transplanted kidneys in the iliac fossa presents an opportunity for evaluation [21]. Therefore, we assessed a group of renal transplant recipients (RTRs) with AA-A for kidney stiffness measurement (KSM) of the transplanted kidneys. As a control group for kidney measurements, we studied RTRs who underwent transplantation due to conditions other than amyloidosis, and the known underlying diseases, including DM, hypertension, and chronic glomerulonephritis due to various causes, were recorded.

The Istanbul Faculty of Medicine Research Ethics Committee approved the study protocol (02.03.2021–107645 for the liver study, and 02.03.2021–107634 for the kidney study), and written informed consent was obtained from all participants in accordance with the Declaration of Helsinki principles.

### Definition of organ involvement in amyloidosis

The biopsy site for the histopathologic confirmation of amyloidosis was selected based on the primary organ affected during the clinical course. If a biopsy from the affected organ could not be obtained, subcutaneous adipose tissue, gastrointestinal mucosa, or gingiva were chosen due to their easy accessibility. The diagnosis of amyloidosis was confirmed using Congo red staining, and the type of amyloidosis (AA and AL) was determined through immunohistochemistry. Liver involvement by AA-A or AL-A was defined by either biopsy findings or by meeting the criteria established by Gertz and colleagues. These criteria include a total liver span > 15 cm in the absence of heart failure or an alkaline phosphatase level > 1.5 times the institutional upper limit of normal, which were developed for assessing liver involvement in AL-A [18].

Cardiac amyloid involvement was evaluated with echocardiography (Echo), performed by one experienced cardiologist (PKO). Gertz and colleagues’ definition of heart involvement in AL-A includes only the increase of the mean left ventricular or septal wall thickness (CSWT) (> 12 mm) in the absence of hypertension or other potential causes of left ventricular hypertrophy [18, 19]. To be able to improve the definition, we also evaluated the presence of at least one of the following three additional appropriate Echo findings: (i) decreased ejection fraction, ii) increased granular echogenicity and/or amyloid valvulopathy, iii) presence of left ventricular diastolic dysfunction (defined as in Mitter et al. study [22]), after exclusion of the differential diagnoses, in patients with confirmed amyloidosis in other organs.

We used the “Kidney Disease: Improving Global Outcomes (KDIGO)” definitions for chronic kidney disease (eGFR < 60 ml/min/1.73 m^2^ for 3 months or more) (CKD), and end-stage renal disease (ESRD, eGFR < 15 ml/min/1.73 m^2^, or requirement of renal replacement therapy such as kidney transplantation or dialysis) [23].

Patients with a positive biopsy result and chronic diarrhea with findings of malabsorption were considered to have gastrointestinal amyloidosis.

### Laboratory evaluation

All participants were evaluated for liver function (alanine aminotransferase [ALT], aspartate aminotransferase [AST], alkaline phosphatase [ALP], gamma-glutamyl transferase [GGT], kidney function (serum creatinine and urine protein/creatinine ratio), and cardiac function (high-sensitivity troponin T [normal value < 14 pg/mL] and N-terminal pro-brain natriuretic peptide [pro-BNP, normal value < 125 pg/mL]). Standard laboratory assessments, including hemogram and acute phase reactants such as C-reactive protein (CRP), during the attack-free period, were also performed. Fibrosis-4 (FIB-4) score and AST to platelet ratio index (APRI) were also calculated for each patient in all groups [24]. All patient FIB-4 scores were categorized by advanced fibrosis risk: low-risk (FIB-4 < 1.3); indeterminate-risk (FIB-4:1.3 – 2.67); and high-risk (FIB-4 > 2.67) using the risk thresholds established for non-alcoholic fatty liver disease (NAFLD) [25].

### FibroScan evaluation

Transient elastography measurements with FibroScan were performed by an experienced gastroenterologist (BC) under single-blind setting. LS and KS were expressed in kilopascals (kPa), which range from 1.5 to 75.0. The probe utilizes pulse-echo ultrasound to follow the propagation of the shear wave to measure velocity (m/s) and provide LS and KS measurements in representative volumes of liver and transplanted kidney tissues [26]. Ten valid measurements were taken from each patient to ensure reliable results for fibrosis and maintain the technical quality of the scan for the liver and kidney separately [27]. The ratio of the interquartile range (IQR) of LS and KS to the median (IQR/M) was calculated as an indicator of variability and reliability. The patients who had IQR/M > 30%, reflecting high variability, were considered failures, and those patients were excluded from the study [27, 28]. LSMs were categorized as kPa < 7 normal (S0-1 stiffness), S2 stiffness between 7 and 9.4 kPa, S3 stiffness between 9.5 and 12.4 kPa, S4 stiffness ≥ 12.5 kPa. Advanced stiffness was also defined as ≥ 9.5 kPa (S3 and S4 stiffness) [13].

The same methodology used for liver measurements was applied to assessing transplanted kidneys, utilizing the "M" probe. The "XL" probe was employed when prompted by the system.

### Genetic evaluation

Genetic analysis was routinely performed in all FMF patients, followed in a reference center when possible, and the results for the *MEFV* gene exon 10 missense variants (M694V, M694I, M680I (G/C), M680I (G/A), K695R, R761H, V726A, A744S) were recorded from their patient charts [29]. FMF patients were grouped and compared with each other according to the presence of two exon 10 (homozygous and compound heterozygous) or one exon 10 (heterozygous) *MEFV* variant, and M694V homozygosity, or the presence of other *MEFV* variants (M680I, V726A, etc.) according to the differences in their penetrance and potential risk for amyloidosis. Exon 2 (E148Q) and exon 3 (P369S) *MEFV* variants were not taken into consideration due to their lower penetrance and/or uncertain significance in patients with FMF [29, 30].

### Statistical analysis

The SPSS (Statistical Package for the Social Sciences) program v29.0 (IBM, Armonk, NY, USA) was used for the statistical analysis of the data. In descriptive statistics, discrete and continuous numerical variables were expressed as mean, ± standard deviation, median, and IQR. Categorical variables were expressed as number of cases and (%). Cross-table statistics were used to compare categorical variables first for the overall significance using the Kruskal–Wallis test, which was followed by pairwise comparisons with the Kolmogorov–Smirnov test to identify the contributing items when there was a significant difference between groups.

Normally distributed parametric data were compared with Student's t-test; non-parametric data that did not meet normal distribution were compared with Mann–Whitney. Multivariate analysis was performed by logistic regression. Correlation analysis was performed using the Pearson or Spearman method according to normality distribution. Sensitivity and specificity calculations were performed using receiver operating characteristic (ROC) analysis. p < 0.05 value was considered statistically significant.

## Results

### Liver stiffness measurement

**Fig. 1:**
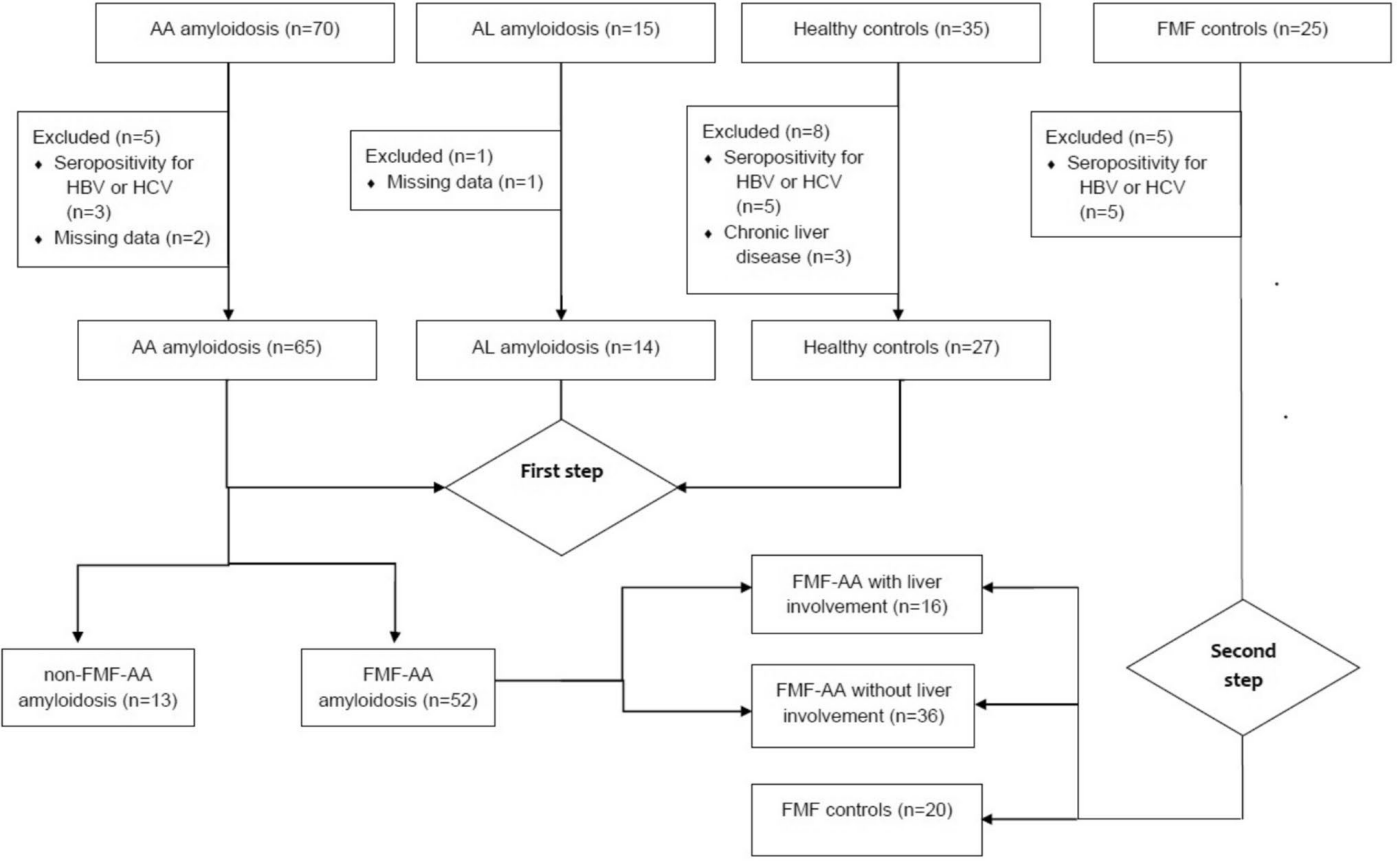
Flowchart of the participants in the study

We evaluated 70 AA-A, 15 AL-A, 25 FMF, and 35 HC patients for LS analysis. After excluding 3 AA-A patients due to positive serology for hepatitis B or C, 3 patients (2 AA-A, 1 AL-A) due to missing data, 5 FMF patients due to seropositivity for hepatitis B or C, and 8 HC patients due to a history of DM or chronic hepatitis, a total of 126 participants were included (Fig. 1).

The most frequent cause of AA-A was FMF (80%, 52/65). Clinical and laboratory features of the patients with AA-A and their comparison with AL-A are given in Table 1 and Supplementary Table 1, respectively. The male/female ratio, BMI values, and frequency of DM did not differ among all groups. Only the average age of the patients with AL-A was higher than the average age in the other groups. Development of ERSD was higher in AA-A compared to AL-A; however, the frequency of the heart, bone marrow, biopsy-proven liver involvement, and the extent of amyloid burden were higher in the AL-A group compared to the frequencies in the AA-A group (Supplementary Table 1).

**Table 1:** Clinical and laboratory features of patients with AA amyloidosis

Variables	Results (n = 65)
Age (years), mean (SD)	47 (12)
Sex, male, n (%)	38 (5)
Etiology of amyloidosis, n, (%)	
Familial Mediterranean Fever	52 (80)
Ankylosing spondylitis	4 (6)
Idiopathic	3 (5)
DADA2 disease	2 (3)
Behçet’s disease	1 (1.5)
Crohn’s disease	1 (1.5)
Rheumatoid arthritis	1 (1.5)
Takayasu arteritis	1 (1.5)
FMF-AA patients	
Onset age of FMF (years), median (IQR)	7 (5)
Diagnosis age of FMF (years), mean (SD)	25 (13)
Family history of FMF (n = 53, %)	29 (55)
Duration of underlying disease (years), mean (SD)	27 (12)
Diagnosis age at amyloidosis (years), mean (SD)	35 (15)
Duration of amyloidosis (years), median (IQR)	10 (13)
Family history of amyloidosis (n = 56, %)	20 (36)
Cardiac parameters	
Ejection Fraction, mean (SD)	65 (8)
Cardiac Septal Wall Thickness (mm), mean (SD)	11.5 (3)
Left Ventricular Wall Thickness (cm), mean (SD)	4.4 (0.5)
Pro-BNP (pg/mL), median (IQR)	758 (3971)
Troponin (pg/mL), median (IQR)	18 (68)
Renal parameters	
Proteinuria at admission (g/day), median (IQR)	3.3 (5)
Creatinine at admission (mg/dL), median (IQR)	0.8 (0.4)
Proteinuria at the last visit (g/day), median (IQR)	0 (1.8)
Creatinine at the last visit (mg/dL), median (IQR)	1.2 (0.8)
CRP at the last visit (mg/L), median (IQR)	3 (6)
Colchicine dose (mg/day), median (IQR)	1.5 (0.9)
B-DMARD treatment, n, (%)	44 (68)
Duration of b-DMARD (months), median (IQR)	39 (55)
Anti-IL-1 treatment, n, (%)	36 (55)
Other b-DMARD treatment, n, (%)	11 (17)
Organ involvement of AA amyloidosis, n, (%)	
Renal	63 (96.9)
Gastrointestinal system	18 (27.7)
Heart	13/55 (23.6)
Liver (biopsy-proven)	3 (4.6)
Bone marrow	3 (4.6)
Thyroid	2 (3.1)
Overall development of CKD, n, (%)	45 (69)
Overall development of ESRD, n, (%)	34 (52)
Renal transplantation, n, (%)	25 (39)

*SD* Standard deviation, *IQR* Interquartile range, *FMF* Familial Mediterranean Fever, *FMF-AA* Familial Mediterranean Fever associated AA amyloidosis, *kPa* Kilopascal, *CRP* C-reactive protein, *b-DMARD* Biological disease-modifying anti-rheumatic drug, *ALT* Alanine aminotransferase, *AST* aspartate aminotransferase, *ALP* Alkaline phosphatase, *GGT* Gama-glutamyl transferase, *DADA2* Deficiency of Adenosine Deaminase 2, *CKD* Chronic kidney disease, *ESRD* End-stage renal disease, other b-DMARD treatment Tumor necrosis alpha inhibitors, secukinumab, tocilizumab

First, we compared the LS measurements in patients with AL-A, AA-A, and HC. Median (IQR) LS was 9.8 (11) in AL-A, 6.4 (5.4) in AA-A, and 4.7 (1.7) in HC. Using Kruskal -Wallis analysis, there was a significant difference between the groups (asymptotic significance, two-sided, p < 0.0001). LS was significantly higher in patients with AA-A and AL-A compared with HC (Table 2). The LS value was numerically higher in AL-A than in AA-A, but this difference did not reach statistical significance.

**Table 2:** Comparison of liver stiffness and clinical and laboratory parameters between patients with amyloidosis and control groups

Variables	AA Amyloidosis (n = 65)	AL Amyloidosis (n = 14)	Healthy Controls (n = 27)	P^1^ (OR)	P^2^ (OR)	P^3^ (OR)
Age (years), mean (SD)	47 (12)	58 (15)	45 (16)	0.2	***0.017***	0.006
Sex, male, n (%)	38 (59)	6 (43)	17 (55)	0.7	0.2	0.5
Diabetes Mellitus, n (%)	5 (8.3)	2 (14.3)	3 (9.7)	0.8	0.6	0.9
Body Mass Index (kg/m^2^), mean (SD)	26 (2.3)	25 (2)	26 (5)	0.7	0.3	0.7
Liver stiffness (kPa), median (IQR)	6.4 (5.4)	9.8 (11)	4.7 (1.7)	** < *0.001***	0.16	** < *0.001***
S0-1 stiffness, n (%)	34 (52.3)	7 (53.8)	27 (100)	** < *0.001 (4.4)***	0.3	** < *0.001 (4)***
S2 stiffness, n (%)	14 (21.5)	1 (8)	0	***0.009 (2.6)***		0.2
S3 stiffness, n (%)	7 (10.8)	2 (15.4)	0	0.08		***0.04 (2)***
S4 stiffness, n (%)	10 (15)	3 (23)	0	***0.03 (4.7)***		***0.001 (11.9)***
Advanced stiffness (≥ 9.5 kPa), n (%)	17 (26)	7 (54)	0	***0.003 (8.7)***	***0.048 (3.9)***	** < *0.001 (17.6)***
FIB-4 score, median (IQR)	0.97 (0.9)	1.3 (1)	0.7 (0.5)	***0.005***	0.14	0.6
FIB-4 score (≥ 1.3), n (%)	22 (34.4)	7 (50)	3 (12.5)	0.03 (4.2)	***0.25***	***0.02 (5.2)***
FIB-4 score (≥ 2.67), n (%)	4 (6.3)	1 (7)	1 (4)	0.9	0.7	0.7
APRI, median (IQR)	0.25 (0.2)	0.24 (0.2)	0.16 (0.08)	***0.002***	0.4	***0.02***
Platelet levels (10^3^ mcg/L), median (IQR)	232 (83)	209 (89)	271 (70)	***0.035***	0.3	0.03
ALT (U/L), median (IQR)	19 (17)	15 (7)	17 (13)	0.4	0.15	***0.02***
AST (U/L), median (IQR)	20 (17)	8 (48)	18.5 (8)	0.058	0.08	0.08
ALP (U/L), median (IQR)	97 (65)	103 (54)	67 (22)	** < *0.001***	0.7	***0.002***
GGT (U/L), median (IQR)	18 (18)	24 (61)	14 (14)	0.065	0.084	0.2
Liver amyloid involvement, n (%)	24 (37)	5 (35.7)		0.9		

P^1^: Comparison of AA amyloidosis and healthy control

P^2^: Comparison of AA and AL amyloidosis

P^3^: Comparison of AL amyloidosis and healthy control

*SD* Standard deviation, *IQR* Interquartile range, *FMF* Familial Mediterranean Fever, *kPa* Kilopascal, *FIB-4* Fibrosis-4 index, *APRI* AST to platelet ratio index, *ALT* Alanine aminotransferase, *AST* aspartate aminotransferase, *ALP* Alkaline phosphatase, *GGT* Gammaglutamyl transferase, *OR* Odds ratio

Bold-italic values are statistically significant (p < 0.05)

We then analyzed the amyloidosis patients with liver involvement as defined by Gertz et al. The frequency of liver involvement was comparable between AA-A and AL-A patients (37% vs 35.7%; p = 0.9). S0-1 stiffness was lower, S4 and advanced stiffness were higher in patients with AA-A and AL-A than in HC. In comparison of stiffness categories, only the frequency of advanced stiffness was higher in patients with AL-A than in those with AA-A (Table 2). The median (IQR) LS values were also higher in patients with biopsy-proven liver AA-A involvement than those without (12.9 [25] vs 6.2 [4] kPa; p = 0.013) (Supplementary Table 2).

In the second step, we compared the LS measurements in FMF-AA patients with and without liver involvement and also in FMF patients without findings of amyloidosis. Kruskal–Wallis analysis of the overall group comparison revealed no significant difference between the groups (asymptotic significance, two-sided, p = 0.356). Among FMF-AA, 16 patients (30.7%) were identified as having liver involvement, as defined by *Gertz *et al. Median LS was 6.8 kPa in patients with FMF-AA with liver involvement, 5.7 kPa in those without liver involvement, and 7.15 kPa in the FMF control group, and there was no statistically significant difference between the groups. Only S4 stiffness was higher in patients with FMF-AA with and without liver involvement than in the FMF control group (Table 3).

**Table 3:** Comparison of clinical and laboratory findings among patients with Familial Mediterranean Fever (FMF)-Associated Amyloidosis with and without liver involvement and FMF control group

Variables	FMF-AA without liver involvement (n = 36)	FMF-AA with liver involvement (n = 16)	FMF control group (n = 20)	p^1^ (OR)	p^2^ (OR)	p^3^ (OR)
Age (years), mean (SD)	41.7 (10)	50.9 (22)	43.4 (11.7)	***0.008***	0.08	0.6
Age at diagnosis of amyloidosis (years), median (IQR)	26.5 (15)	35 (30)	NA	***0.009***	NA	NA
Duration of amyloidosis (years), median (IQR)	12 (12)	10.6 (13.8)	NA	0.4	NA	NA
Sex, male, n (%)	20 (55.6)	9 (56.3)	10 (50)	0.9	0.7	0.7
Diabetes Mellitus, n (%)	2/34 (6)	1/14 (7)	2/18 (11)	0.9	0.7	0.7
Body Mass Index (kg/m^2^), mean (SD)	25.4 (0.5)	26.9 (4.1)	26.8 (4.3)	0.3	0.95	0.3
Liver stiffness (kPa), median (IQR)	5.7 (3.6)	6.8 (6.6)	7.15 (4.6)	0.13	0.3	1
S0-1 stiffness, n (%)	22 (61)	8 (50)	9 (45)	0.3	0.1	0.2
S2 stiffness, n (%)	9 (25)	2 (12.5)	7 (35)		0.1	0.4
S3 stiffness, n (%)	2 (5.6)	3 (18.8)	4 (20)		0.9	0.09
S4 stiffness, n (%)	3 (8.3)	3 (18.8)	0		***0.04 (4)***	***0.04 (4)***
Advanced stiffness (≥ 9.5 kPa), n (%)	5 (14)	6 (37.5)	4 (20)	0.054	0.2	0.2
FIB-4 score, median (IQR)	0.85 (0.74)	1.3 (0.9)	0.77 (0.6)	***0.007***	***0.01***	0.2
FIB-4 score (≥ 1.3), n (%)	11 (30.6)	6 (40)	3 (19)	0.6	0.2	0.2
FIB-4 score (≥ 2.67), n (%)	1 (2.8)	2 (12.5)	0	0.1	0.1	0.5
APRI, median (IQR)	0.28 (0.15)	0.3 (0.23)	0.26 (0.18)	0.3	0.3	0.9
ALT (U/L), median (IQR)	20 (22)	19.5 (11)	28.5 (36)	0.8	0.1	0.1
AST (U/L), median (IQR)	22 (14)	21 (21)	22.5 (18)	0.7	0.9	0.9
ALP (U/L), median (IQR)	92 (43)	159 (112)	79 (55)	** < *0.001***	***0.001***	0.7
GGT (U/L), median (IQR)	16 (15.5)	28.5 (43.5)	17 (26)	***0.006***	0.1	0.3
Creatinine at admission (mg/dL), median (IQR)	0.7 (0.4)	1.3 (0.7)	NA	***0.007***	NA	NA
EF, median (IQR)	68 (5)	65 (10)	NA	0.064	NA	NA
CSWT (mm), mean (SD)	11.3 (2.6)	12.1 (2.8)	NA	0.3	NA	NA
Two exon 10 variants, n (%)	30/32 (93.7)	12/15 (80)	10/11 (91)	0.4	0.7	0.7
M694V homozygous, n (%)	24/32 (75)	9/15 (60)	2/11 (18)	0.3	***0.03 (4.6)***	***0.03 (4.6)***
Heart involvement, n (%)	9 (28)	7 (44)	NA	0.3	NA	NA
GIS involvement, n (%)	7 (19.4)	6 (37.5)	NA	0.2	NA	NA
Overall development of CKD, n (%)	21 (58.3)	15 (93.8)	NA	***0.01 (6.5)***	NA	NA
Overall development of ESRD, n (%)	17 (47)	12 (75)	NA	0.06	NA	NA
Colchicine dose, median (IQR)	1.5 (0.5)	1 (1)	NA	***0.007***	NA	NA
B-DMARD usage, n (%)	25 (69.4)	5 (69)	NA	1	NA	NA

P^1^: Comparison of FMF-AA with and without liver involvement

P^2^: Comparison of FMF-AA with liver involvement and FMF control group

P^3^: Comparison of FMF-AA without liver involvement and FMF control group

*SD* Standard deviation, *IQR* Interquartile range, *FMF*: Familial Mediterranean Fever, *FMF-AA* FMF-associated amyloidosis, *kPa* Kilopascal, *FIB-4* Fibrosis-4 score, *APRI* AST to platelet ratio index, *ALT* Alanine aminotransferase, *AST* aspartate aminotransferase, *ALP* Alkaline phosphatase, *GGT* Gama-glutamyl transferase, *EF*: Ejection fraction, *CSWT* Cardiac septal wall thickness, *OR* Odds ratio, *NA* Not applicable

Bold-italic values are statistically significant (p < 0.05)

In FMF-AA, the LS value (median 18.6 vs 5.7 kPa), cardiac and GIS involvements, amyloid burden (≥ 2 organs), S3, S4, and advanced stiffness were higher in patients who had one exon 10 variant compared to those who had two variants. S3, S4, and advanced stiffness, GIS, and cardiac involvement, amyloid burden (≥ 3 organs), and mean CSWT were higher in patients with the other MEFV variants compared to those with the homozygous M694V variant (Supplementary Table 3).

### Relationship between LS and clinical and laboratory parameters

FIB-4 score, APRI, ALT, and AST levels were lower in AA-A patients compared to those in AL-A. FIB-4 score, APRI, AST, and ALP levels were higher, and ALT levels were lower in patients with AA-A compared to those in FMF patients. FIB-4 score, APRI, and ALP levels were higher in AA-A patients compared to those in HC (Table 2). FIB-4 scores were also higher in those with one exon 10 variant compared to the scores in those with two exon 10 variants among the FMF-AA patients (Supplementary Table 3).

FIB-4 score, APRI, AST, ALP, and GGT levels were correlated with LS in AA-A, AL-A, and FMF-AA patients. Proteinuria and troponin levels at the last visit correlated with the LS only in patients with AA-A. Patient age, age of onset of the underlying disease, age at the diagnosis of amyloidosis, and CSWT had a mild correlation with the LS in patients with AA-A and FMF-AA (Table 4).

**Table 4:** Correlation between liver stiffness and clinical and laboratory parameters

Variables	AA amyloidosis		AL Amyloidosis		FMF-AA		Non-FMF-AA	
	r	P value	R	P value	r	P value	r	P value
Age (years)	*0.354*	***0.004***	0.075	0.7	*0.374*	***0.006***	0.241	0.4
Body mass index (kg/m^2^)	-0.046	0.8	-0.06	0.8	0.017	0.9	-0.213	0.5
FIB-4 score	*0.504*	** < *0.001***	*0.536*	***0.048***	*0.584*	** < *0.001***	0.105	0.7
APRI	*0.485*	** < *0.001***	*0.579*	***0.03***	*0.566*	** < *0.001***	0.166	0.6
ALT (U/L)	0.143	0.26	0.09	0.8	0.124	0.4	0.231	0.45
AST (U/L)	*0.341*	***0.006***	*0.542*	***0.046***	*0.329*	***0.02***	0.395	0.2
ALP (U/L)	*0.437*	** < *0.001***	*0.645*	***0.013***	*0.322*	***0.024***	*0.799*	***0.001***
GGT (U/L)	*0.506*	** < *0.001***	*0.752*	***0.002***	*0.306*	***0.033***	*0.946*	** < *0.001***
Onset age of clinical symptoms (years)	*0.303*	***0.043***	NA	NA	*0.313*	***0.049***	0.853	0.066
Duration of underlying disease (years)	0.028	0.85	-0.52	0.067	0.03	0.85	-0.099	0.85
Diagnosis age of amyloidosis (years)	*0.384*	***0.003***	0.17	0.56	*0.391*	***0.007***	0.392	0.2
Duration of amyloidosis (months)	-0.038	0.78	-0.47	0.088	0.062	0.68	-0.256	0.4
Proteinuria at admission (g/day)	-0.083	0.64	0.354	0.2	-0.112	0.6	0.585	0.1
Creatinine at admission (mg/dL)	0.077	0.66	0.27	0.35	0.078	0.7	-0.203	0.6
Proteinuria at the last visit (g/day)	-0.052	0.7	*0.655*	***0.02***	-0.055	0.7	0.1	0.8
Creatinine at the last visit (mg/dL)	-0.056	0.7	0.35	0.2	-0.086	0.55	-0.026	0.9
CRP at the last visit (mg/L)	-0.068	0.6	0.2	0.5	-0.048	0.74	0.324	0.3
Colchicine dose (mg/day)	-0.038	0.8	NA	NA	0.068	0.64	NA	NA
Ejection fraction	0.056	0.7	-0.15	0.6	0.126	0.4	-0.241	0.43
Cardiac septal wall thickness (mm)	*0.319*	***0.012***	0.383	0.2	*0.314*	***0.03***	0.365	0.22
Left ventricular wall thickness (cm)	-0.178	0.17	0.184	0.5	0.194	0.2	-0.210	0.5
Pro-BNP levels (pg/mL)	0.226	0.53	0.434	0.072	0.286	0.5	0.268	0.5
Troponin levels (pg/mL)	0.555	0.2	*0.740*	***0.001***	0.073	0.84	0.401	0.25

*SD* Standard deviation, *IQR* Interquartile range, *FMF* Familial Mediterranean Fever, *CRP* C-reactive protein, *FIB-4* Fibrosis-4 index, *APRI* AST to platelet ratio index, *ALT* Alanine aminotransferase, *AST* Aspartate aminotransferase, *ALP* Alkaline phosphatase, *GGT* Gamma-glutamyl transferase, *NA* Not applicable

Bold-italic values are statistically significant (p < 0.05)

### Factors associated with LS

The median patient age and age at diagnosis of amyloidosis were higher (p = 0.008 and p = 0.009, respectively), and the median colchicine dose (1 vs 1.5 mg/day) was lower in patients with liver involvement than in those without, according to the *Gertz *et al*.* criteria (p = 0.007). Higher age at the diagnosis of amyloidosis (p = 0.04, Odds ratio [OR] = 1.12, 95% Confidence interval [CI]: 1.006–1.249), and ALP levels (p = 0.046, OR = 1.02, 95% CI: 1.0–1.04) were associated with liver AA-A in biopsy-proven patients in multivariate analysis (Supplementary Table 2). Median creatinine levels and overall frequency of CKD were higher in patients with liver involvement than those without in FMF-AA patients (p = 0.007 and p = 0.01, respectively). FIB-4 score, ALP, and GGT levels were higher in patients with liver involvement than those without in FMF-AA in univariate analysis (p = 0.007, p < 0.001, and p = 0.006, respectively) (Table 3). In multivariate analysis, only higher age at amyloidosis diagnosis was associated with liver involvement in patients with FMF-AA (p = 0.032, OR: 1.104, 95% CI: 1.009–1.209).

**Fig. 2:**
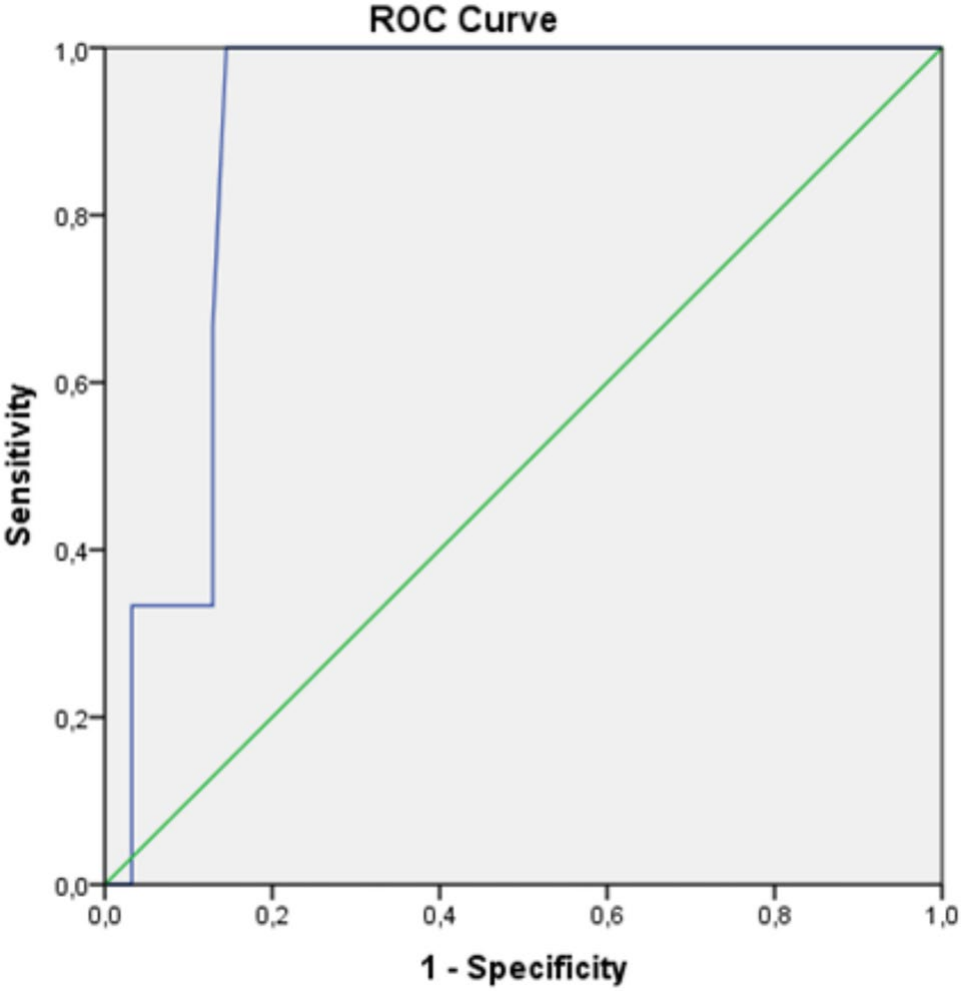
The ROC curve of liver stiffness in patients with AA amyloidosis according to biopsy-proven liver involvement

**Fig. 3:**
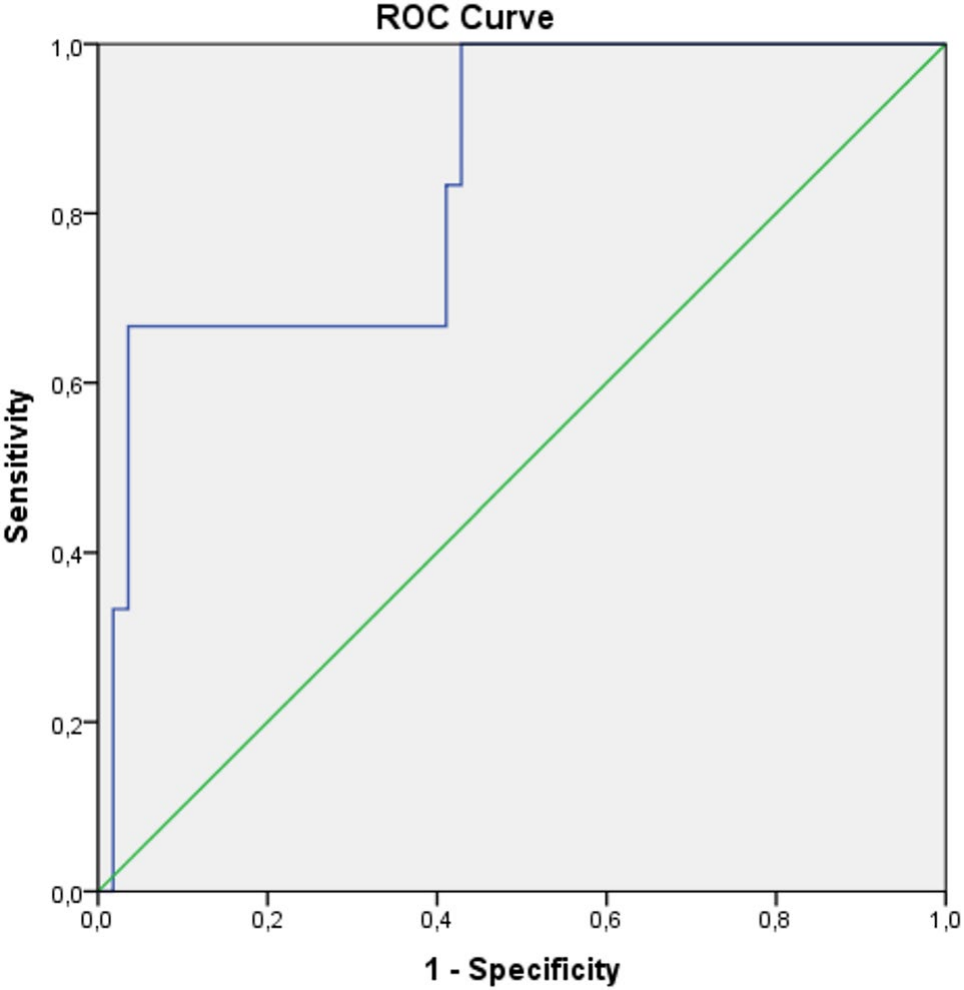
The ROC curve of liver stiffness in patients with AA amyloidosis according to the definition of the *Gertz *et al*.* criteria

The ROC analysis provided a cut-off value of 12.05 kPa for LS with 100% sensitivity and 85.5% specificity (Likelihood ratio [LR] = 6.9; area under the curve [AUC] = 0.901; 95% CI: 0.81–0.99) in patients with the biopsy-proven liver AA-A (Fig. 2). Two cut-off values, 18.1 kPa (LR = 18.7) and 12.05 kPa (LR = 4.7), with a sensitivity of 66.7% for both, specificity of 96.4% and 85.7%, respectively, p = 0.006, AUC = 0.842, 95% CI: 0.68–1) were calculated for liver AA-A according to the *Gertz *et al*.* criteria (Fig. 3). A cut-off value of 17.15 kPa (LR = 12) for LS with 100% sensitivity and 91.7% specificity, (p = 0.0045, AUC = 0.958, 95% CI: 0.85–1) was calculated in patients with AL-A with the same criteria (Supplementary Fig. 1); but no cut-off value was observed in AL-A patients with biopsy-proven liver involvement (Supplementary Fig. 2).

### KS measurements

Nineteen AA-A and 16 control group (kidney control group) patients were included in the KS analyses, and no participants were excluded. Patient age, sex, BMI, donor type, donor sex, donor age, history of rejection, and graft loss did not differ between the two groups. The prevalence of DM, serum creatinine, and proteinuria levels were lower in AA-A compared to the kidney control group. Most of the patients with AA-A (73.7%) had homozygous M694V. The KS values were comparable between the AA-A and kidney control groups (median 15.8 vs 19.8 kPa) (Supplementary Table 4). Thirteen patients had a recurrence of AA-A in the transplanted kidney (68.4%), defined by the reappearance of proteinuria and confirmed with a biopsy. Median KS was significantly higher in patients with AA-A recurrence in the transplanted kidney than those without (29.3 vs 10.9 kPa, Table 5). Although median stiffness was higher in patients with a history of rejection in amyloidosis and kidney control groups, they did not reach statistical significance (18.7 [12] vs 14.8 [15.8]; p = 0.3 and 24.2 [36] vs 15.8 [37]; p = 0.18).

**Table 5:** Comparison of clinical and laboratory features between patients with renal transplantation recipients due to AA amyloidosis who had amyloidosis recurrence and those who did not

Variables	Total	Recurrence of amyloidosis		
		No (n = 13)	Yes (n = 6)	P value (odds ratio)
Age (years), median (IQR)	48 (22)	47 (17)	50 (27)	1
Sex, male, n (%)	13 (68.4)	9 (69.2)	4 (66.7)	1
Duration of amyloidosis (months), median (IQR)	206 (89)	220 (99)	163 (203)	0.08
Onset age of FMF (years), median (IQR)	5.5 (2)	6 (2)	5 (6)	0.3
Duration of FMF (years), median (IQR)	41 (20)	41 (14)	45 (24)	0.6
Age at diagnosis of FMF (years), median (IQR)	21 (20)	23.5 (21)	21 (11)	0.6
Age at diagnosis of amyloidosis (years), median (IQR)	28 (17)	27.5 (17)	28 (20)	1
Duration of renal transplantation (months), median (IQR)	145 (137)	144 (110)	123 (50)	0.7
Donor age (years), median (IQR)	46 (20)	46 (38)	46 (15)	0.8
Donor sex, male, n (%)	12 (92.3)	4 (30.8)	1 (16.7)	***0.037 ***^***Ɨ***^*** (6.6)***
Donor type, alive, n (%)	12 (92.3)	6 (85.7)	6 (100)	1
BMI (kg/m^2^), median, (IQR)	24.9 (1.2)	25.3 (3.9)	24.7 (4.4)	0.3
Kidney stiffness (kPa), median (IQR)	15.8 (15.8)	10.9 (7.7)	29.3 (18.9)	0.003*
Liver stiffness (kPa), median (IQR)	5.45 (2.8)	5.4 (2.7)	5.9 (8.9)	0.4
History of rejection, n (%)	3 (15.8)	2 (15.4)	1 (16.7)	1
Creatinine at the last visit (mg/dL), median (IQR)	1.4 (0.6)	1.4 (0.7)	1.7 (0.5)	0.24
CRP at the last visit (mg/L), median, (IQR)	2.7 (4.4)	1.3 (4.1)	3.5 (13.9)	0.3
Proteinuria at the last visit (g/day), median, (IQR)	0 (0)	0 (0)	0 (2.8)	0.4
ALT (U/L), median, (IQR)	20 (12)	20 (8)	23.5 (33)	0.7
AST (U/L), median, (IQR)	20 (13)	22 (12)	17.5 (24)	0.6
ALP (U/L), median, (IQR)	94.5 (49)	93 (38)	101 (116)	0.9
GGT (U/L), median, (IQR)	18 (13)	18 (11)	16.5 (75)	1
Organ distribution, n (%)				
Liver	0			
Gastrointestinal (n = 17)	7 (41.2)	4 (33.3)	3 (60)	0.6
Heart (n = 17)	6 (35.5)	4 (33.3)	2 (40)	0.8
B-DMARD, n (%)	16 (84.2)	10 (76.9)	6 (100)	0.5
Immunosuppressives, n (%)				
Calcineurin inhibitors	17 (89.5)	13 (100)	4 (66.7)	0.09
m-TOR inhibitors	2 (10.5)	0	2 (33.3)	

*IQR* Interquartile range, *FMF* Familial Mediterranean Fever, *kPa* Kilopascal, *CRP* C-reactive protein, *BMI* Body mass index, *b-DMARD* Biological disease-modifying anti-rheumatic drug, *ALT* Alanine aminotransferase, *AST* aspartate aminotransferase, *ALP* Alkaline phosphatase, *GGT* Gamma-glutamyl transferase

^*^Mann–Whitney U test

^Ɨ^ Fisher’s Exact test

Bold-italic values are statistically significant (p < 0.05)

**Fig. 4:**
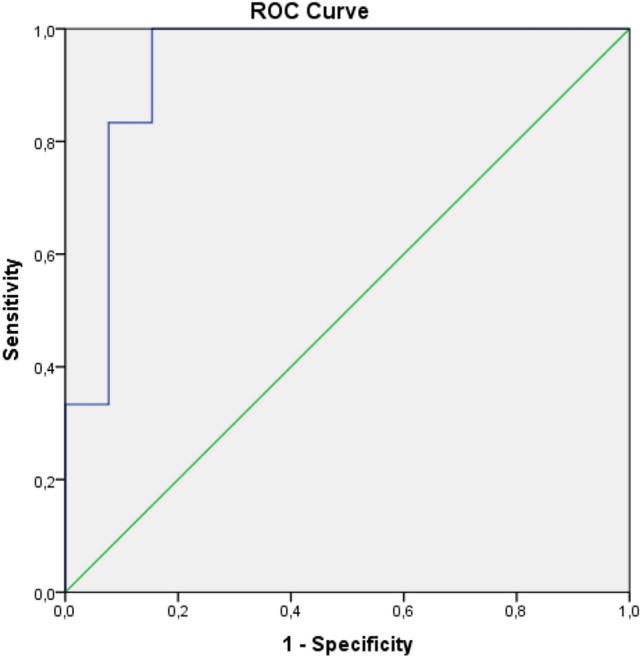
The ROC curve of kidney stiffness in patients with AA amyloidosis in renal transplant recipients

In multivariate analysis, only KS was associated with AA-A recurrence in patients with RTRs (p = 0.031; OR = 1.18, 95% CI: 1.015–1.362). In the ROC analysis, two cut-off values were calculated for the recurrence of AA-A in the transplanted kidney as 17.65 kPa with 100% sensitivity and 84.6% specificity (LR = 6.5), and 24.55 kPa with 83.3% sensitivity, 92.3% specificity (LR = 10.8, AUC = 0.936, p = 0.003, 95% CI: 0.82–1.0) (Fig. 4).

In patients with FMF-AA, median KS was higher in those patients with one MEFV exon 10 variant compared to those with two exon 10 variants, and the KS values tended to be higher in patients with other MEFV variants compared to those with homozygous M694V (33.4 vs 14.1, and 29.3 vs 14.75 kPa; respectively) (Supplementary Table 5). There was no correlation between the KS values and patient age, disease duration, duration of renal transplantation, donor age, BMI, LS, creatinine, CRP, and proteinuria levels in patients with AA-A and kidney CG.

## Discussion

This study aimed to evaluate the potential clinical utility of FibroScan regarding the involvement of amyloid deposition in patients with AA-A. Our study offers novel insights into organ involvement in AA-A by utilizing a non-invasive technique that has not previously been explored in this detail. Moreover, the inclusion of well-defined control groups—patients with AL amyloidosis, healthy controls, and individuals with FMF—allowed for a more precise comparison of liver stiffness measurements across different conditions. This comparative approach distinguishes our findings from earlier reports on AA amyloidosis and strengthens the relevance of our results [31–34]. LS was significantly higher in patients with AA-A and AL-A compared with HC, but not in AA-A patients with liver involvement compared to AA-A with no liver involvement. Median KS was significantly higher in patients with AA-A recurrence in the transplanted kidney than in those without. In the *Gertz *et al*.* study, the prevalence of liver involvement was 25% (19 of 77 patients) in patients with AL-A, and the authors also defined hepatic AL involvement non-invasively by the combination of hepatomegaly and increased ALP levels (≥ 1.5 times) [18, 35]. However, these criteria have not been validated yet in AA-A. To our knowledge, this is the first study to demonstrate higher liver stiffness (LS) values obtained by TE using FibroScan in a large group of patients with AA-A. The comparative results with AL-A, along with the observation of higher LS values corresponding to the extent of organ involvement, may suggest a potential association between LS values and amyloid burden. However, as a preliminary study, these findings are not conclusive. Further research involving similar measurements alongside SAP scintigraphy or biopsy-proven documentation of organ involvement is necessary to validate these results.

Although tissue biopsy is still the gold standard in the diagnosis of amyloidosis, along with the documentation of organ involvement, tissue sampling also has some risks, including bleeding, which makes the procedure nearly impossible in some patients. New methods beyond SAP scintigraphy have been tested in recent years for detecting liver amyloid involvement, such as SWE  , and MRE , reflecting higher LS in patients with amyloidosis [9, 10]. Besides these results, extremely high LS was revealed in three case reports by TE measurements in patients with AA-A and AL-A [36, 37]. Deposition of amyloid fibrils may increase the rigidity of the organ parenchyma, which results in higher stiffness values [16]. A recent study indicated higher LS values in patients with liver AL-A involvement than those without (median 14.4 kPa vs. 8.1 kPa; p = 0.001), similar to our results [38]. Furthermore, this study revealed higher LS values in patients with AL-A liver involvement compared to AL-A without liver involvement and transthyretin amyloidosis patients with cardiac involvement as a control group, since liver involvement is not a feature of transthyretin amyloidosis. Although cut-off values of LS differed according to the gold-standard method (SAP scintigraphy or the *Gertz *et al. criteria) in the former study, it supports the use of FibroScan in the diagnosis of liver amyloid involvement, as in our study. Moreover, *Loustaud-Ratti *et al.’s study revealed higher LS in patients with AL-A compared to the patients with right-sided heart failure, HCV, and myeloma [16]. The authors also reported a cut-off value of 17.3 kPa with 92.4% sensitivity and 63.6% specificity for hepatic AL-A involvement [16]. In our study, the LS values were higher both in AA-A and AL-A compared to HC, which was compatible with the former studies. Our study yielded a similar cut-off value (17.15 kPa) compared to the previous one in patients with AL-A, with higher sensitivity and specificity. On the other hand, the observation of a lower LS value in AA-A compared to AL-A in our study was a new finding, which may be due to higher amyloid accumulation in patients with AL-A compared to AA-A.

In the current study, increased LS values were also observed in patients with liver involvement defined by using the *Gertz *et al*.* criteria. Liver involvement in AL-A was previously found to be associated with the widespread involvement of other organs, such as the heart and kidney, which was consistent with our results. Moreover, a higher frequency of hepatomegaly and elevated ALP levels in patients with AL-A compared to those with AA-A was also reported by *Levy *et al. [39]. In another large study, liver amyloid involvement was detected by SAP scintigraphy in 54% of AL-A and 18% of AA-A patients [40]. The authors also concluded that hepatic amyloid deposition is associated with a higher amyloid burden and occurs late in AA-A compared to AL-A, which is consistent with our results. ALP levels were higher in patients with AA-A and AL-A compared to the other groups and correlated with the LS values in patients with AA-A (FMF or non-FMF related), and AL-A in our study. GGT levels tended to be higher both in patients with AA-A and AL-A compared to the patients with HC, and they also significantly correlated with the LS values in patients with AA-A (FMF and non-FMF related) and AL-A. Increased cholestatic enzyme levels (ALP and GGT) were more suggestive than transaminases for liver amyloid deposition, as previously shown [40, 41]. We herein demonstrated higher FIB-4 scores and APRI, which indicate CLD/fibrosis in patients with AA-A and AL-A compared to the patients with FMF and HC, and these scores were also found to be higher in patients with FMF compared to HC. These results were compatible with the findings of a moderate correlation between FIB-4 score, APRI, and LS values in patients with AA-A, FMF-AA (but not in the relatively smaller subgroup of non-FMF-AA), and AL-A. Also, several studies revealed an association between FMF and CLD, such as NAFLD and cryptogenic cirrhosis [42–44]. Higher LS and liver enzymes, as well as APRI and FIB-4 scores in patients with FMF compared to HC, indicate that FMF patients may face a higher risk of CLD compared to HC, even in the absence of amyloidosis.

Increased prevalence of hepatitis and/or cirrhosis in patients with FMF may be due to the direct pathogenic role of *MEFV* variants, colchicine toxicity, or higher prevalence of NAFLD in patients with FMF. The lack of association between colchicine dose and cirrhosis makes colchicine toxicity unlikely [42]. Additionally, findings of lower LS values in patients with FMF-AA compared to non-FMF-AA suggest that colchicine or IL-1 blockade may even have a protective role. Lower LS values, as well as lower prevalence of heart, GIS, and liver involvement in patients with two exon 10 variants, including M694V homozygous patients, compared to the patients with heterozygous exon 10 variants or non-M694V variants, may indicate that additional factors other than penetrant *MEFV* variants may affect the risk of liver involvement and amyloid burden in AA-A. The result of the current study was interesting and challenging because of the close association between the penetrant *MEFV* variants and higher disease severity as well as increased risk of AA-A [45, 46]. Although the penetrant *MEFV* variants are associated with the development of amyloidosis more frequently and at an earlier age [47], the current study indicates that it may not be responsible for more widespread amyloidosis in patients with FMF-AA. On the other hand, FMF patients with heterozygous or less penetrant *MEFV* variants may experience delays in diagnosis or receive lower doses of colchicine. Therefore, the risk of developing AA-A constitutes a complex issue, and it can be speculated that additional factors beyond the *MEFV* variants may contribute to the overall risk of amyloidosis. Furthermore, the contribution of non-*MEFV* genetic factors may be more important in the development of amyloidosis in FMF-AA patients with less penetrant *MEFV* variants, and these additional factors may be associated with more extensive disease in the multifactorial nature of AA-A. Further studies are needed to clarify the potential role of the *MEFV* and non-*MEFV* variants, treatment, and environmental factors on the amyloid burden in patients with AA.

While heart involvement in AL-A is already well-documented [48], it has traditionally been considered rare in AA-A [8]. In one study, evaluation of cardiac amyloidosis in 114 patients with increased septal wall thickness, cardiac amyloidosis was identified in 50 cases, of which one was attributed to AA-A [49]. Higher LS may also be associated with a higher frequency of cardiac involvement in patients with AL-A. However, there was a weak correlation between CWST and LS in AA-A, and the lack of correlation among AL-A patients did not support this hypothesis. Furthermore, the absence of a relationship between cardiac involvement and LS in AL-A patients was also shown in another study [16]. In our study, we established a higher frequency of cardiac involvement in AA-A, which was more than one-fifth of our patients, even though its frequency is lower compared to the frequency in AL-A. Higher cardiac involvement was also established in our previous study [47] and case reports [50]. The results of the current study suggest that mild cardiac involvement may be overlooked in an important proportion of patients with AA-A. Moreover, these results were remarkable despite using a more comprehensive definition of cardiac amyloidosis in our study compared to the criteria used by *Gertz *et al*.* [18]. Although kidney involvement and development of ESRD were lower in AL-A, bone marrow, liver, and heart involvement and amyloidosis burden (≥ 3 organs) were higher compared to AA-A in our study.

Although information about baseline KS by the TE method is scarce, some studies investigated the utility of TE instead of kidney biopsy for chronic allograft nephropathy in patients with RTRs [51, 52]. In the *Lukenda *et al*.* study, mean allograft stiffness was 32.2 kPa and positively correlated with the extent of interstitial fibrosis on kidney biopsy (r = 0.727, p < 0.001) and negatively correlated with e-GFR values (r = -0.640, p < 0.001) [51]. These findings were confirmed by the *Arndt *et al*.* study [52]. In our study, although median KS was similar between the amyloidosis and control groups, it was significantly higher in patients who had AA-A recurrence in the transplanted kidney than those who did not (29.3 vs 10.9; p < 0.001). Furthermore, a cut-off value of 24.55 kPa was found to provide high diagnostic accuracy in our study. These findings suggest that TE may be used for screening AA-A recurrence instead of more invasive procedures, such as graft biopsy, in patients with RTRs. Further studies in a larger group, including comparison studies with those patients with biopsy-confirmed diagnosis, are needed to support these findings.

This study has some strengths and limitations. Initially, the investigation of a relatively large group of AA-A patients with different characteristics was an important asset of the study, improving the reliability of the findings, including the relationship of these findings with clinical and biochemical parameters. Providing cut-off values according to different organ definitions was also the strength of the study. Also, a higher frequency of patients with the *MEFV* variant results in patients with FMF-AA, which allowed us to make the genotype–phenotype correlation analysis. The primary limitations of the study were the small number of AA amyloidosis patients with confirmatory liver biopsies and the absence of reliable data for assessing amyloid burden, such as SAP scintigraphy results. On the other hand, matching the groups for the confounding factors affecting the development of CLD, such as age, gender, and DM frequency, and screening the participants in all groups for viral hepatitis also helped the evaluation of the findings more effectively.

## Conclusion

The current study revealed that the average values of LS were higher in patients with AL-A and AA-A compared to the values in FMF and HC, and these values correlated with the serum ALP and GGT levels as well as with the number of organs involved. The findings of this study also supported the potential of the TE method using FibroScan in the detection of the recurrence of amyloidosis in the transplanted kidneys. Higher LS and KS values were observed in non-FMF-AA compared to FMF-AA, which may be associated with the favorable effects of colchicine and other effective treatments in FMF. Further studies would be helpful to establish the potential use of the TE method in the follow-up of patients for the deposition of amyloid fibrils in the liver and transplanted kidney.

## Supplementary Information

Below is the link to the electronic supplementary material.

Supplementary file1 (DOCX 62 KB)

## Data Availability

Study datasets are available from the corresponding author upon reasonable request.

## Abbreviations

TE: Transient elastography
FMF: Familial mediterranean fever
CLD: Chronic liver disease
HC: Healthy controls
LS: Liver stiffness
RTR: Renal transplant recipient
KS: Kidney stiffness
