# Supplementary material for: Transient elastography measurements of the liver and transplanted kidney in patients with AA amyloidosis: a cross-sectional comparative study
Source: Rheumatol Int. 2025 Jul 1;45(7):162. doi: 10.1007/s00296-025-05906-3 (PMC12213854; doi:10.1007/s00296-025-05906-3)

**Supplementary Tables**

| Variables | AA amyloidosis (n=65) | AL amyloidosis (n=14) | p value (odds ratio) |
| --- | --- | --- | --- |
| Organ involvement, n (%) |  |  |  |
| Renal | 63 (96.9) | 12 (85.7) | 0.083 |
| Gastrointestinal | 18 (27.7) | 5 (35.7) | 0.5 |
| Heart | 20/61 (32.8) | 11 (78.6) | ***0.002 (10)*** |
| EF<50 | 4 (6.8) | 2 (14.3) | 0.3 |
| CSWT>12 mm | 20/61 (32.8) | 11 (78.6) | ***0.002 (9.8)*** |
| Granular echogenicity | 14/60 (23.3) | 6 (42.9) | 0.1 |
| LVDD | 3/22 (13.6) | 6/10 (60.3) | ***0.013 (7.3)*** |
| Bone marrow | 3 (4.6) | 10 (71.4) | ***<0.001 (37.4)*** |
| Liver (biopsy-proven) | 3 (4.6) | 5 (35.7) | ***<0.001 (12.2)*** |
| Chronic kidney disease, n (%) | 45 (69.2) | 8 (57) | 0.38 |
| End-stage renal disease, n (%) | 34 (52.3) | 3 (21.4) | ***0.036 (4.4)*** |
| Amyloidosis burden (≥2 organs), n (%) | 28 (43.1) | 9 (64.3) | 0.15 |
| Amyloidosis burden (≥3 organs), n (%) | 11 (16.9) | 8 (57) | ***0.001 (10.2)*** |

**Supplementary Table 1.** Comparison of clinical and laboratory features of patients with AA and AL amyloidosis

EF: Ejection fraction, CSWT: Cardiac septal wall thickness, LVDD: Left ventricular diastolic dysfunction

Bold-italic values are statistically significant (p<0.05)

**Supplementary Table 2**. Univariate and multivariate analysis of clinical and laboratory parameters in patients with biopsy-proven liver AA amyloidosis involvement

| Variables | Biopsy-proven liver involvement | | | |
| --- | --- | --- | --- | --- |
|  | **Univariate analysis** | | | **Multivariate analysis** |
|  | **Yes (n=3)** | **No (n=62)** | **P value (odds ratio)** | **P value (OR) [95% CI)** |
| Age (years), median (IQR) | 69 (7.2) | 45 (17) | ***0.007*** |  |
| Gender, male, n (%) | 2 (66.7) | 36 (94.7) | 0.8 |  |
| Diagnosis age at amyloidosis (years), median (IQR) | 57 (11.5) | 30 (21) | ***0.015*** | ***0.04 (1.12) [1.006-1.249]*** |
| Duration of amyloidosis (years), median (IQR) | 72 (80) | 10.3 (12.7) | 0.4 |  |
| CSWT (mm), median (IQR) | 14 (2) | 11 (4) | 0.07 |  |
| FIB-4 score, median (IQR) | 2.06 (0.5) | 0.93 (0.8) | ***0.04*** |  |
| APRI score, median (IQR) | 0.25 (0.2) | 0.25 (0.17) | 0.7 |  |
| Liver stiffness (kPa), median (IQR) | 12.9 (25) | 6.2 (4) | ***0.013*** |  |
| ALT (U/L), median (IQR) | 15 (20) | 19.5 (16.75) | 0.5 |  |
| AST (U/L), median (IQR) | 23 (25) | 20 (16.4) | 0.8 |  |
| ALP (U/L), median (IQR) | 180 (86.7) | 96 (53) | ***0.009*** | ***0.046 (1.02) [1.0-1.04]*** |
| GGT (U/L), median (IQR) | 44 (381) | 17.5 (16.75) | 0.4 |  |
| Etiology of amyloidosis, n (%) |  |  |  |  |
| FMF-AA | 0 | 52 (84) | ***0.007 (12.6)*** |  |
| Non-FMF-AA | 3 (100) | 10 (16) |  |  |
| Liver stiffness (kPa), n (%) |  |  |  |  |
| <7 | 0 | 34 (55) | 0.1 |  |
| ≥7 | 3 (100) | 28 (45) |  |  |
| Liver stiffness (kPa), n (%) |  |  |  |  |
| <9.5 | 0 | 48 (77) | ***0.016 (8.9)*** |  |
| ≥9.5 | 3 (100) | 14 (23) |  |  |
| Liver stiffness (kPa), n (%) |  |  |  |  |
| <12.5 | 1 (33.3) | 54 (87) | 0.06 |  |
| ≥12.5 | 2 (66.7) | 8 (13) |  |  |
| Exon 10 copy, n (%) |  |  |  |  |
| One | NA | 8 (15.7) |  |  |
| Two | NA | 43 (84.3) |  |  |
| MEFV status, n (%) |  |  |  |  |
| M694V homozygous | 0 | 33/52 (58) | 0.085 |  |
| Others | 3 (100) | 24/52 (42) |  |  |

SD: Standard deviation, IQR: Interquartile range, OR: Odds ratio, CI: Confidence interval, FMF: Familial Mediterranean Fever, kPa: Kilopascal, CSWT: Cardiac septal wall thickness, FIB-4: Fibrosis-4 score, APRI: AST to platelet ratio index, ALT: Alanine aminotransferase, AST: Aspartate aminotransferase, ALP: Alkaline phosphatase, GGT: Gamma-glutamyl transferase, NA: Not available

Bold-italic values are statistically significant (p<0.05)

**Supplementary Table 3**: Comparison of clinical and laboratory features of patients with Familial Mediterranean Fever-associated amyloidosis according to MEFV gene status

| Variables | One exon 10 variant (n=4) | Two exon 10 variants (n=43) | p value (odds ratio) | M694V homozygotes (n=33) | Other MEFV variants (n=14) | p value (odds ratio) |
| --- | --- | --- | --- | --- | --- | --- |
| Age (years), mean (SD) | 60 (6.5) | 43.2 (11.2) | ***0.008*** | 42.6 (11) | 49.4 (13) | 0.098 |
| Sex, male, n (%) | 3 (75) | 24 (55.8) | 0.4 | 19 (57.6) | 17 (63) | 0.7 |
| Diagnosis age at amyloidosis (years), median (IQR) | 48.5 (26) | 29 (14) | ***0.01*** | 28 (12) | 38.5 (23) | ***0.02*** |
| Duration of amyloidosis (years), median (IQR) | 9 (15.4) | 12 (11.6) | 0.5 | 13.3 (12.5) | 8.5 (9.8) | 0.1 |
| Organ involvement of amyloidosis, n (%) |  |  |  |  |  |  |
| Renal | 4 (100) | 41 (95.3) | 1 | 32 (97) | 26 (96.3) | 1 |
| Gastrointestinal system | 3 (75) | 9 (21) | ***0.02 (5.4)*** | 5 (15.2) | 7 (50) | ***0.025***  ***(6.3)*** |
| Heart | 3 (75) | 10/39 (26) | ***0.04 (4.2)*** | 5/30 (16.7) | 9 (64.3) | ***0.002 (10)*** |
| Liver (biopsy-proven) | NA | NA |  | 0 | 3 (11.1) | 0.085 |
| Liver (biopsy+Gertz criteria) | 2 (50) | 12/42 (28.6) | 0.4 | 9 (27.3) | 6 (43) | 0.3 |
| Bone marrow | 0 | 1 (2.3) | 1 | 1 (3) | 1 (3.7) | 1 |
| Thyroid | 1 (12.5) | 1 (2.7) | 0.3 | 1 (3) | 1 (3.7) | 1 |
| Amyloidosis burden (≥2 organs), n (%) | 4 (100) | 14 (33.3) | ***0.009 (6.8)*** | 10 (30) | 9 (64) | ***0.05 (4.7)*** |
| Amyloidosis burden (≥3 organs), n (%) | 1 (25) | 4 (9.3) | 0.4 | 1 (3) | 4 (28.6) | ***0.023 (6.7)*** |
| Chronic kidney disease, n (%) | 3 (75) | 28 (65.1) | 0.7 | 21 (63.6) | 19 (70.4) | 0.8 |
| End-stage renal disease, n (%) | 3 (75) | 22 (51.2) | 0.3 | 18 (54.5) | 12 (44.4) | 0.4 |
| Liver stiffness (kPa), median (IQR) | 18.6 (48) | 5.7 (3.4) | ***0.001*** | 5.6 (3.5) | 8.95 (24.3) | 0.1 |
| S0-1 stiffness (kPa≥7), n (%) | 0 | 27 (64.3) | ***0.01 (9.5)*** | 20 (61) | 7 (50) | 0.5 |
| S2 stiffness, n (%) | 0 | 9 (21.4) | 0.3 | 9 (27.3) | 0 | ***<0.001 (10)*** |
| S3 stiffness, n (%) | 2 (50) | 2 (4.8) | ***<0.001 (20)*** | 2 (6.1) | 3 (21.4) | ***0.03 (6.2)*** |
| S4 stiffness (kPa≥12.5), n (%) | 2 (50) | 4 (9.5) | ***0.02 (9.5)*** | 2 (6.1) | 4 (28.6) | ***0.034 (4.5)*** |
| Advanced stiffness (kPa≥9.5), n (%) | 4 (100) | 6 (14.3) | ***<0.001 (15.8)*** | 4 (12.1) | 7 (50) | ***0.009 (7.9)*** |
| FIB-4 score, median (IQR) | 1.8 (1.9) | 0.93 (0.82) | ***0.034*** | 0.92 (0.74) | 1.5 (1.3) | 0.054 |
| FIB-4 score (≥1.3), n (%) | 3 (75) | 12/41 (29.3) | 0.064 | 9 (27.3) | 7 (54) | 0.088 |
| FIB-4 score (≥2.67), n (%) | 1 (25) | 2/41 (4.9) | 0.1 | 1 (3) | 2 (15.4) | 0.2 |
| APRI, median (IQR) | 0.4 (1) | 0.3 (0.17) | 0.4 | 0.26 (0.16) | 0.33 (0.38) | 0.2 |
| ALT (U/L), median (IQR) | 27 (56.3) | 20 (13.3) | 0.3 | 20.5 (15.75) | 19.5 (26.8) | 0.6 |
| AST (U/L), median (IQR) | 31 (50.8) | 20.5 (14.3) | 0.2 | 20 (12.8) | 22 (24.3) | 0.4 |
| ALP (U/L), median (IQR) | 153.5 (100) | 96 (49) | 0.2 | 93 (46) | 119 (91.3) | 0.4 |
| GGT (U/L), median (IQR) | 32 (171) | 17 (15) | ***0.007*** | 17 (14) | 27 (16) | 0.2 |
| Ejection Fraction, mean (SD) | 67.8 (2) | 63.7 (9.1) | 0.4 | 63.5 (9.1) | 65.2 (8.2) | 0.6 |
| Cardiac septal Wall Thickness (mm), mean (SD) | 15.3 (0.5) | 11.1 (2.4) | ***0.002*** | 10.85 (2.1) | 12.9 (2.9) | ***0.013*** |
| Left Ventricular Wall Thickness (cm), mean (SD) | 4.4 (0.3) | 4.3 (0.6) | 0.8 | 4.4 (0.5) | 4.2 (0.6) | 0.3 |
| Proteinuria at admission (g/day), median (IQR) | 4 (3.9) | 3 (6.7) | 0.8 | 2.8 (6.2) | 4.3 (5.4) | 0.8 |
| Creatinine at admission (mg/dL), median (IQR) | 2 (1.5) | 0.8 (0.4) | ***0.02*** | 0.7 (0.5) | 0.95 (0.4) | 0.08 |
| Proteinuria at the last visit (g/day), median (IQR) | 2.4 (5.7) | 1.1 (0.9) | 0.4 | 0 (0.53) | 0.25 (3.3) | 0.1 |
| Creatinine at the last visit (mg/dL), median (IQR) | 1.6 (2.7) | 1.1 (0.71) | 0.2 | 1.1 (0.7) | 1.26 (1.2) | 0.1 |
| CRP at the last visit (mg/L), median (IQR) | 3.4 (7.9) | 2.8 (6.3) | 0.7 | 2.6 (5.8) | 3.6 (11.5) | 0.6 |
| Colchicine dose (mg/day), median (IQR) | 1 (0.4) | 1.5 (0.5) | 0.3 | 1.5 (0.5) | 1 (0.6) | 0.1 |
| B-DMARD treatment, n (%) | 3 (75) | 32 (74.4) | 0.7 | 24 (72.7) | 18 (66.7) | 0.6 |
| Duration of b-DMARD treatment (months), median (IQR) | 36.5 (36.5) | 37.5 (53) | 1 | 34 (52) | 40.5 (39) | 0.9 |

SD: Standard deviation, IQR: Interquartile range, FMF: Familial Mediterranean Fever, kPa: Kilopascal, CRP: C-reactive protein, b-DMARD: Biological disease-modifying anti-rheumatic drug, FIB-4: Fibrosis-4 index, APRI: AST to platelet ratio index, ALT: Alanine aminotransferase, AST: Aspartate aminotransferase, ALP: Alkaline phosphatase, GGT: Gamma-glutamyl transferase, NA: Not available

Bold-italic values are statistically significant (p<0.05)

**Supplementary Table 4**. Baseline clinical and laboratory characteristics of renal transplant recipients for kidney stiffness evaluation

| Variables | AA Amyloidosis (n=19) | Control group (n=16) | p value (odds ratio) |
| --- | --- | --- | --- |
| Age (years), median, (IQR) | 48 (22) | 51 (11) | 0.4 |
| Sex, male, n (%) | 13 (68.4) | 11 (68.8) | 1 |
| Duration of renal transplantation (months), median, (IQR) | 126 (77) | 145 (137) | 0.5 |
| Duration of renal transplantation (months), median, (IQR) | 145 (137) | 126 (77) | 0.5 |
| Donor age (years), median, (IQR) | 46 (20) | 56 (28) | 0.3 |
| Donor type, alive, n (%) | 12/13 (92.3) | 11 (68.8) | 0.2 |
| BMI (kg/m^2^), median, (IQR) | 24.9 (1.2) | 28 (5.75) | 0.4 |
| Diabetes mellitus, n (%) | 0 | 5 (31.3) | ***0.013 (6.9)*** |
| Kidney stiffness (kPa), median, (IQR) | 15.8 (15.8) | 19.8 (34) | 0.46 |
| Liver stiffness (kPa), median, (IQR) | 5.45 (2.8) | 6.1 (4.5) | 0.9 |
| History of rejection, n (%) | 3 (15.8) | 5 (33.3) | 0.4 |
| Graft loss^ĸ^, n (%) | 0 | 2 (13.3) | 0.18 |
| Creatinine at the last visit (mg/dL), median, (IQR) | 1.4 (0.6) | 1.95 (1.8) | ***0.015*** |
| CRP at the last visit (mg/L), median, (IQR) | 2.7 (4.4) | 1.95 (10.3) | 0.6 |
| Proteinuria at the last visit (g/day), median, (IQR) | 0 (0) | 0.9 (2.4) | ***<0.001*** |
| Hematuria at the last visit, n (%) | 2 (10.5) | 2 (12.5) | 1 |

IQR: Interquartile range, BMI: Body mass index, CRP: C-reactive protein

^ĸ^Requirement for dialysis or transplantation

Bold-italic values are statistically significant (p<0.05)

**Supplementary Table 5:**  Comparison of clinical and laboratory features of patients with renal transplant recipients due to AA amyloidosis according to the MEFV gene status

| Variables | Pathogenic Exon 10 MEFV Variants | | | MEFV Variant Status | | |
| --- | --- | --- | --- | --- | --- | --- |
|  | **One variant (n=3)** | **Two variants (n=15)** | **p-value** | **M694V homozygous (n=14)** | **Others**  **(n=4)** | **p-value** |
| Age (years), median (IQR) | 57 (7.2) | 47 (16) | 0.16 | 47 (18) | 54 (23) | 0.5 |
| Sex, male, n (%) | 3 (100) | 9 (60) | 0.5 | 9 (64.3) | 3 (75) | 0.7 |
| Duration of amyloidosis (months), median (IQR) | 156 (99) | 211 (72) | 0.2 | 206 (84) | 201 (230) | 0.7 |
| Duration of FMF (years), median (IQR) | 52 (9.5) | 38 (17) | 0.1 | 41 (18) | 46.5 (22) | 0.3 |
| Diagnosis age of FMF (years), median (IQR) | 24.5 (5) | 21 (20) | 0.6 | 22 (21) | 21 (9.6) | 0.95 |
| Diagnosis age of amyloidosis (years), median (IQR) | 36.5 (2.1) | 26 (16) | 0.18 | 27 (15) | 35 (16) | 0.9 |
| Kidney stiffness (kPa), median (IQR) | 33.4 (10) | 14.1 (15) | ***0.027*** | 14.75 (15.5) | 29.3 (25.3) | 0.08 |
| Liver stiffness (kPa), median (IQR) | 5.9 (3.5) | 5.4 (2.8) | 0.7 | 5.4 (2.7) | 5.3 (5.4) | 0.7 |
| FIB-4 score, median (IQR) | 1.7 (1.2) | 0.87 (0.93) | ***0.04*** | 0.96 (0.92) | 1.47 (2.4) | 0.2 |
| APRI, median (IQR) | 0.31 (0.65) | 0.2 (0.18) | 0.056 | 0.22 (0.17) | 0.3 (1) | 0.3 |
| Creatinine at the last visit (mg/dL), median (IQR) | 1.5 (0.2) | 1.4 (0.7) | 1 | 1.4 (0.7) | 1.45 (0.4) | 0.95 |
| CRP at the last visit (mg/L), median (IQR) | 11 (14) | 2.7 (3.7) | 0.3 | 2.9 (3.7) | 5.7 (23.6) | 0.9 |
| Proteinuria at the last visit (g/day), median (IQR) | NA | NA |  | 0.57 (0.3) | NA |  |

IQR: Interquartile range, FMF: Familial Mediterranean Fever, kPa: Kilopascal, CRP: C-reactive protein, NA: Not available

Bold-italic values are statistically significant (p<0.05)

**Supplementary Figures**

**Supplementary Figure 1**. ROC curve of liver stiffness in patients with AL amyloidosis according to the definition of *Gertz et al.* criteria


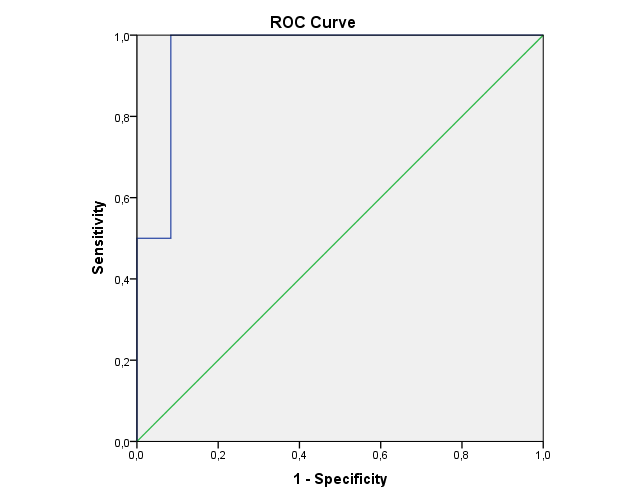


**Supplementary Figure 2**. The ROC curve of liver stiffness in patients with AL amyloidosis according to biopsy-proven liver involvement


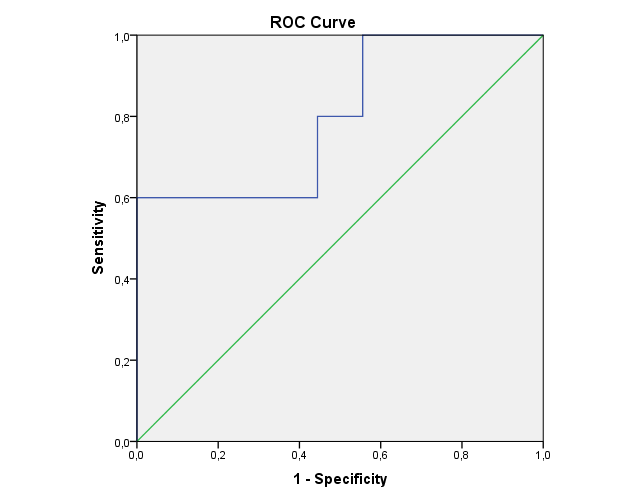

Supplement: Supplementary file 1 — Supplementary file1 (DOCX 62 KB) [file 296_2025_5906_MOESM1_ESM.docx]
